# Mathematical modeling of the hematocrit influence on cerebral blood flow in preterm infants

**DOI:** 10.1371/journal.pone.0261819

**Published:** 2021-12-28

**Authors:** Irina Sidorenko, Varvara Turova, Esther Rieger-Fackeldey, Ursula Felderhoff-Müser, Andrey Kovtanyuk, Silke Brodkorb, Renée Lampe

**Affiliations:** 1 Chair of Mathematical Modeling, Mathematical Faculty, Technical University of Munich, Garching, Germany; 2 Research Unit for Pediatric Neuroorthopedics and Cerebral Palsy of the Buhl-Strohmaier Foundation, Orthopedic Department, School of Medicine, Klinikum rechts der Isar, Technical University of Munich, Munich, Germany; 3 Department of Pediatrics, School of Medicine, Klinikum rechts der Isar, Technical University of Munich, Munich, Germany; 4 Neonatology, Pediatric Intensive Care, Pediatric Neurology, Department of Pediatrics I, University Hospital Essen, University Duisburg-Essen, Essen, Germany; 5 Neonatology Department, Munich Clinic Harlaching, Munich, Germany; Universidade de Lisboa Instituto Superior Tecnico, PORTUGAL

## Abstract

Premature birth is one of the most important factors increasing the risk for brain damage in newborns. Development of an intraventricular hemorrhage in the immature brain is often triggered by fluctuations of cerebral blood flow (*CBF*). Therefore, monitoring of *CBF* becomes an important task in clinical care of preterm infants. Mathematical modeling of *CBF* can be a complementary tool in addition to diagnostic tools in clinical practice and research. The purpose of the present study is an enhancement of the previously developed mathematical model for *CBF* by a detailed description of apparent blood viscosity and vessel resistance, accounting for inhomogeneous hematocrit distribution in multiscale blood vessel architectures. The enhanced model is applied to our medical database retrospectively collected from the 254 preterm infants with a gestational age of 23–30 weeks. It is shown that by including clinically measured hematocrit in the mathematical model, apparent blood viscosity, vessel resistance, and hence the *CBF* are strongly affected. Thus, a statistically significant decrease in hematocrit values observed in the group of preterm infants with intraventricular hemorrhage resulted in a statistically significant increase in calculated *CBF* values.

## Introduction

Due to current knowledge and advances in neonatal care, 90% of preterm infants survive, but up to 50% of very low birth weight infants (< 1500 *g*) develop some sort of permanent neurological impairment caused by injury to the preterm brain [1]. Intraventricular hemorrhage (*IVH*) remains the major complication of the premature birth, especially for very preterm infants with less than 32 weeks gestation (*WG*) [2]. At this age, a specific region containing a highly fragile vessel network, called germinal matrix (*GM*), is still present in the brain [3] and can trigger development of *IVH*. Impaired cerebral autoregulation (cerebral pressure-passivity) in combination with variations in mean arterial pressure (*MAP*) and arterial carbon dioxide partial pressure (*pCO*_*2*_) causes strong disturbances in cerebral blood flow (*CBF*) leading to *IVH* [4]. Therefore, implementation of regular monitoring of *CBF* in clinical routine is an important task for the care of preterm infants. Although several diagnostic techniques, such as near-infrared spectroscopy (*NIRS*) [5], Xenon-133 clearance measurements [6], transcranial Doppler ultrasonography [7], *MRI* based arterial spin labeling (*MRI ASL*) [8], and diffusion correlation spectroscopy (*DCS*) [5], have been developed during last few years, they are still not part of clinical routine monitoring. Therefore, mathematical assessment of the *CBF* can become a promising tool for *CBF* control in preterm infants in order to identify infants at risk.

A recently developed hierarchical cerebrovascular mathematical model [9–11] calculates *CBF* in the immature brain from clinically measured *MAP* and *pCO*_*2*_ using a constant value of apparent blood viscosity. However, the vessel diameter and presence of blood cells suspended in blood plasma, especially red blood cells (*RBCs*), strongly influence the apparent viscosity of blood [12], and hence the resistance of the vessel network and *CBF*. The size of *RBCs* strongly affects the flow properties of blood in tubes with diameter less than 330 *μm* [12]. If *RBC*s are uniformly distributed throughout the vessel volume, their concentration can be measured as the systemic hematocrit (*H*_*SYS*_). In the capillaries, *RBCs* collect near the centerline of the vessel, which leads to the formation of a cell free plasma layer adjacent to the vessel wall and, as a result, to a decreased concentration of *RBCs* known as Fåhraeus effect [13]. This reduced *RBCs* concentration can be described by the tube hematocrit (*H*_*T*_), which is the volume fraction of *RBCs* that are inside the vessel at a given time instant. The net outcome of reduced *RBCs* concentration is a lower apparent viscosity in small arterioles (less than 200 *μm* in diameter) and capillaries, relative to the measured value in large feed arteries [14], which implies the reduction of flow resistance known as Fåhraeus-Lindqvist effect [15]. With decreasing capillary diameter, the apparent blood viscosity exhibits a further strong decrease reaching a minimum at about 6 *μm* [12]. For capillaries with diameters less than 6 *μm* the deformation of the erythrocytes takes place [16] and microvessel resistance can be described by an analytic formula [17] for the hydraulic resistance of capillary.

The purpose of the present work is the enhancement of the mathematical model for *CBF* calculation by a realistic description of apparent blood viscosity with accounting for inhomogeneous hematocrit distribution and its dependence on vessel diameter. For arteries and veins, a phenomenological dependence of the apparent viscosity on the vessel diameter [18, 19] is applied, whereas for capillaries a two-phase fluid model for single-file *RBCs* flow accounting for the deformation of *RBCs* in thin vessels [16, 17, 20] is employed. The performance of the enhanced model is demonstrated using clinical data recorded during regular monitoring of 254 preterm infants with the gestational age of 23–30 weeks. The effect of hematocrit value on vessel resistance and *CBF* as well as on differentiation between preterm infants with and without *IVH* is shown.

## Materials and methods

### Clinical data

Clinical data were obtained from the records of 254 preterm infants treated in the Department of Neonatology at the University Hospital Essen and the Department of Pediatrics of the School of Medicine, Klinikum rechts der Isar, Technical University of Munich. The study was approved by the ethical committee of the University Hospital Essen, University Duisburg-Essen (Ref. 16-7284-BO) and ethical committee of the School of Medicine, Klinikum rechts der Isar, Technical University of Munich (Ref. 364/15). No informed consent from parents was necessary because it was a retrospective study. The clinical records have been collected over 11 years (between 01.2006 and 12.2016) and were fully anonymized before data transfer from the neonatal units to the research group. The gestational age of the sample group ranged from 23 to 30 weeks gestation (*WG*) and a body weight from 335 *g* to 1580 *g*. Preterm infants without *IVH* (118) served as control group and those with *IVH* (136) as affected group. Basic demographic characteristics of the cohort are presented in Table 1, in which continuous variables are expressed as mean and standard deviation, while categorical variables are presented as the number of cases and percentages. *IVH* was diagnosed by serial cranial ultrasound examinations and *IVH* severity was classified according to the Papile grading system [21]. *MAP*, *pCO*_*2*_ and *H*_*SYS*_ were collected as standard routine clinical data during the first 10 days after birth in the control group, and for up to 7 consecutive days before and 3 days after hemorrhage in the affected group. All *pCO*_*2*_ values were taken from the capillary or arterial blood gas analysis. Corresponding *MAP* measurements were taken at the same time as blood gas analysis and corresponding *H*_*SYS*_ values were taken from the last available laboratory record. Since intracranial pressure *P*_*ic*_ could not be recorded in preterm infants, a constant *P*_*ic*_ = 5 *mmHg* [22] was used for numerical calculations for all infants. Statistical analysis of clinical parameters and calculated *CBF* was done using the two-sided Wilcoxon’s rank-sum test for continuous variables and Fisher’s exact test for categorical parameters (MATLAB2020a) with a *p*-value less than 0.05 considered to be statistically significant.

**Table 1:** Basic demographic characteristics of the study cohort.

Parameter	All n = 254 (100%)	No *IVH* n = 118 (100%)	With *IVH* n = 136 (100%)	*p-*value*
**Gestational age [*WG*]**	26.46 ± 2.11	26.68 ±2.17	26.26 ± 2.04	0.13
**Birth weight [*g*]**	864.06 ± 279.10	850.68 ± 252.81	875.66 ± 300.50	0.70
**Male**	122 (48.03%)	48 (40.68%)	74 (54.4%)	0.03
**Twins**	64 (25.19%)	27 (22.88%)	37 (27.21%)	0.55
**Triplets**	31 (12.21%)	16 (13.56%)	15 (11.03%)	0.69
**In Vitro Fertilization**	32 (12.6%)	17 (14.4%)	15 (11.0%)	0.51
**Natural birth**	22 (8.66%)	7 (5.93%)	15 (11.03%)	0.18

*p-value is given for difference between control (no *IVH*) and affected (with *IVH*) groups.

### Modeling of *CBF* in immature brain with germinal matrix

A mathematical model for the calculation of *CBF* [10, 11] in the immature brain was derived from a hierarchical cerebrovascular model for the adult brain [9]. In this model, the cerebral vascular system is divided into 19 levels connected in series according to morphological vessel characteristics. Additionally, each level consists of *m*_*j*_ parallel connected vessels. Thus, the total *CBF* is calculated from Kirchhoff’s law as follows:

$$CBF=\left(MAP-P_{ic}\right)/RES,$$

$$RES=\sum_{j=1}^{19}{RES}_{j}^{level},{RES}_{j}^{level}={RES}_{j}/m_{j}.$$

Here *RES* is the total cerebrovascular resistance, ${RES}_{j}^{level}$ is the resistance of vascular level *j*, and *RES*_*j*_ is the resistance of single vessel on level *j* (*j* = 1…19).

On each level, the number of vessels *m*_*j*_ as well as their lengths *l*_*j*_ and diameters *d*_*j*_ are scaled according to the brain weight of each infant estimated from their birth weight [23]. Furthermore, a vascular response on changes of *MAP* and *pCO*_*2*_ is incorporated into the model through an increase or decrease of vessel diameter (i.e., vasodilation or vasoconstriction).

The presence of germinal matrix is modeled as an additional parallel circuit in the capillary level (*j* = 10). Thus, the total resistance of capillary level ${RES}_{10}^{level}$ is calculated from the resistance of the *GM* capillaries ${RES}_{10}^{GM}$ and the resistance of the rest, non-*GM* brain capillaries ${RES}_{10}^{nGM}$ as:

$${RES}_{10}^{level}={\left({\left({RES}_{10}^{GM}\right)}^{-1}+{\left({RES}_{10}^{nGM}\right)}^{-1}\right)}^{-1},$$

$${RES}_{10}^{GM,nGM}={RES}_{GM,nGM}/m_{GM,nGM}$$

Here *RES*_*GM*_ and *RES*_*nGM*_ are the resistances of the individual *GM* and non-*GM* capillaries. The corresponding number of vessels *m*_*GM*_ and *m*_*nGM*_ are estimated according to the gestational age and brain weight of each infant.

A resistance of individual vessel *RES*_*j*_ is calculated by the application of a micropolar fluid model [24, 25] accounting for the presence of rigid, randomly oriented particles (*RBCs*) suspended in a viscous medium. Simplified analytic expressions for the flow velocity profile and hydraulic resistance are derived using power series expansions, based on the description [26, 27] of steady-state flow of micropolar fluid through a pipe with circular cross-section. In this method, a constant value *μ* = 0.003 *pa* ⋅ *s* of apparent blood viscosity is used. Knowing the resistance of individual vessel and global *CBF*, one can calculate blood flow in individual vessel on level *j* as follows:

$${CBF}_{j}=CBF\cdot {RES}_{j}^{level}/{RES}_{j}.$$

### Accounting for the hematocrit in large vessels

To account for the influence of hematocrit on the resistance of large vessels, the constant blood viscosity is replaced by the apparent blood viscosity *μ*_*a*_, which depends on the vessel diameter and concentration of *RBC*s (hematocrit).

For arteries and veins with diameter *d >* 500 *μm* apparent blood viscosity is an almost constant value, which depends only on hematocrit [28]. However, with decreasing vessel diameter the Fåhraeus-Lindqvist effect [15] takes place, resulting in lower viscosity compared to larger vessels. In order to take this effect into account, the calculation of *RES*_*j*_ is done using “*in vivo* viscosity law” developed in [18] and widely applied in numerical simulations [19, 29, 30]. In this phenomenological relationship, variations of apparent viscosity of blood are described as a function of diameter *d μm* by the following set of formulas:

$$\mu_{a}=\mu_{PL}\left[1+\left(\mu_{0.45}-1\right)\cdot \frac{{\left(1-H_{D}\right)}^{C}-1}{{\left(1-0.45\right)}^{C}-1}\cdot {\left(\frac{d}{d-1.1}\right)}^{2}\right]\cdot {\left(\frac{d}{d-1.1}\right)}^{2},$$

$$\mu_{0.45}=6\cdot exp\left(-0.085\cdot d\right)+3.2-2.44\cdot exp\left(-0.06\cdot d^{0.645}\right),$$

$$C=\left(0.8+e^{-0.075\cdot d}\right)\cdot \left(-1+\frac{1}{1+10^{-11}\cdot d^{12}}\right)+\frac{1}{1+10^{-11}\cdot d^{12}}.$$

Here *μ*_*PL*_ = 0.001 *Pa* ⋅ *s* is the viscosity of plasma [17, 30] and *H*_*D*_ is the discharge hematocrit which is defined by the ratio between red blood cell volume and total blood volume. Experimental measurements have shown [31] that the discharge hematocrit *H*_*D*_ is similar to the systemic hematocrit *H*_*SYS*_ taken from arterioles or venules with diameter 6.98 *μm*. Therefore, in numerical calculations we take discharge hematocrit *H*_*D*_ equal to the clinically measured systemic hematocrit *H*_*SYS*_.

### Accounting for the tube hematocrit in capillaries

In capillaries with diameter *d*_*j*_ *<* 10 *μm* (*j* = 10) the blood plasma moves in a cell-free layer near the wall, whereas *RBC*s travel through the vessel in somewhat of a single file line [13, 17]. The resistance of individual *GM* or non-*GM* capillary *RES*_*GM*,*nGM*_ is calculated by the application of two-phase fluid model with single-file *RBC* flow [17, 20] as:

$${RES}_{GM,nGM}=\rho +\left(\hat{\rho }-\rho \right)H_{T}$$

Here, *H*_*T*_ is a tube hematocrit and *ρ* and $\hat{\rho }$ are defined [17] as:

$$\hat{\rho }=\frac{8l}{\pi }∙{\left(\frac{r_{GM,nGM}^{4}-r_{0}^{4}}{\mu_{PL}}+\frac{r_{0}^{4}}{\mu_{RBC}}\right)}^{-1},$$

$$\rho ={\frac{8l}{\pi }\left(\frac{r_{GM,nGM}^{4}}{\mu_{PL}}\right)}^{-1},$$

$$r_{0}=0.3\mu m+0.8\cdot r_{GM,nGM},$$

$$\mu_{PL}\left(H_{T}\right)=0.001∙\left(16+5H_{T}\right)/15\;Pa\cdot s,$$

$$\mu_{RBC}=0.1\;Pa\cdot s.$$

The reduction of the tube hematocrit *H*_*T*_ in vessels with diameter *d μm* is estimated from the discharge hеmatocrit *H*_*D*_ as [32]:

$$H_{T}/H_{D}=H_{D}+\left(1-H_{D}\right)\cdot \left(1+1.7\cdot e^{-0.35\cdot d}-0.6\cdot e^{-0.01\cdot d}\right).$$

As in the previous section, the discharge hematocrit *H*_*D*_ equals to the clinically measured systemic hematocrit *H*_*SYS*_.

## Results

**Fig 1:**
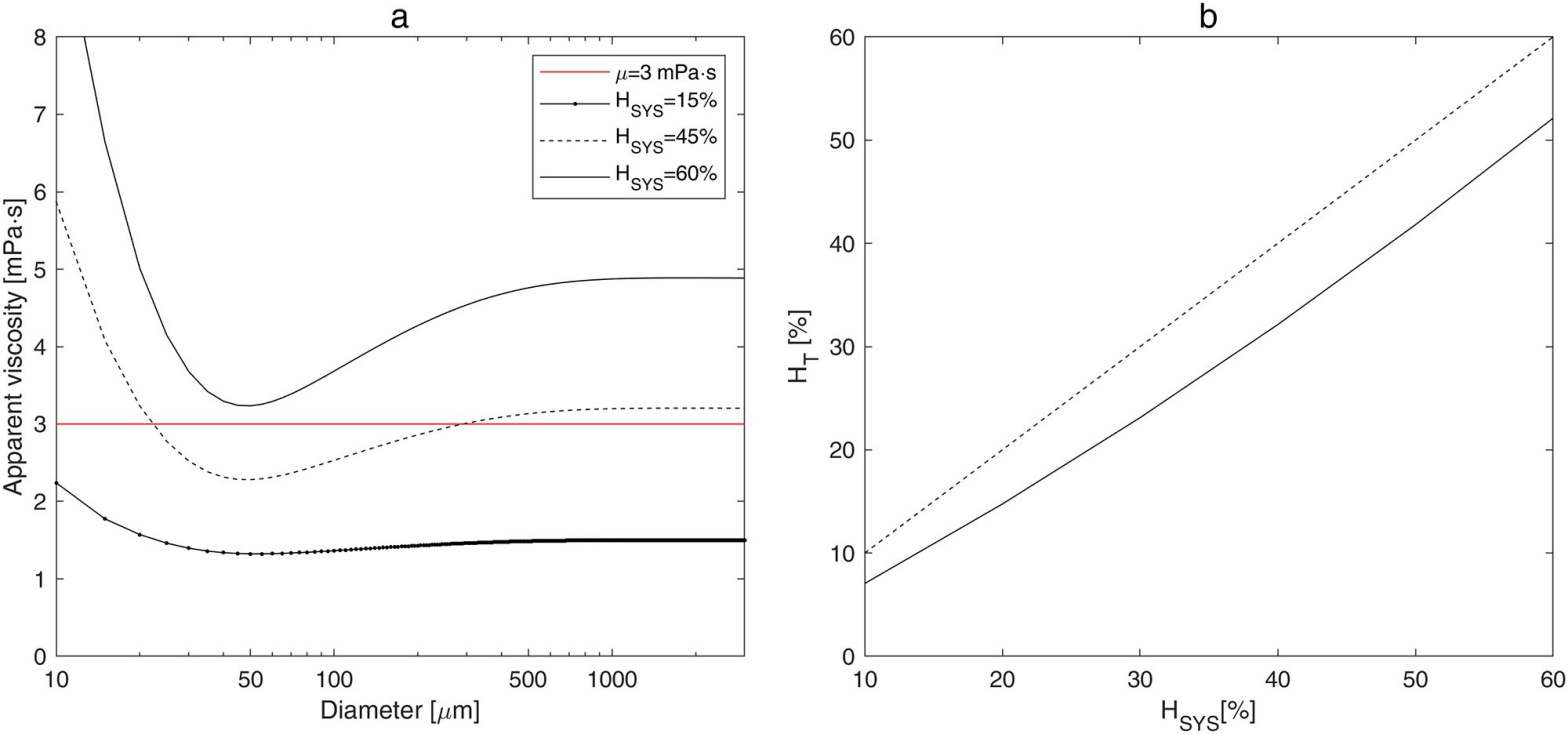
Calculated apparent blood viscosity and tube hematocrit. (a) Calculated apparent blood viscosity versus diameter of vessel for different values of systemic hematocrit. (b) Tube hematocrit versus systemic hematocrit (dashed line *H*_*T*_ *= H*_*SYS*_ is shown for comparison).

The variation of apparent blood viscosity depending on vessel diameter is shown in Fig 1a for several values of systemic hematocrit *H*_*SYS*_. For vessels with *d* > 500 *μm* the apparent blood viscosity is almost independent on vessel diameter (Fig 1a) and for *H*_*SYS*_ = 45% it is close to the constant value *μ* = 0.003 *Pa* ⋅ *s*, which is usually taken for numerical calculations. However, the rise or reduction of systemic hematocrit causes a corresponding increase or decrease of the apparent viscosity (see Table 2). For vessels with *d* < 30 *μm* a strong nonlinear increase of apparent viscosity can be caused both by the increase of hematocrit and decrease of diameter. Thus, for vessels with *d* = 10 *μm* and *H*_*SYS*_ = 60% the apparent viscosity can be more than three times higher than the constant value *μ* = 0.003 *Pa* ⋅ *s* (see Table 2).

**Table 2:** Dependence of computed model parameters on hematocrit.

**Hematocrit [%]**	*H*_*SYS*_		15	45	60
	*H*_*T*_		10.8	36.9	52.1
**Apparent blood viscosity [**10^−3^ *Pa ⋅ s***]**	*μ*	*d* = 10*μm*	2.24	5.87	9.83
		*d* = 500*μm*	1.49	3.1	4.76
**Resistance of individual vessel [**10^16^ *Pa ⋅ s/m*^3^**]** ***WG* = 25**	*RES*_9_		0.789	1.95	3.03
	*RES*_*GM*_		1.03	1.51	1.82
	*RES*_*nGM*_		3.34	5.16	6.33
**Resistance of vascular level [**10^8^ *Pa ⋅ s/m*^3^**]** ***WG* = 25**	${RES}_{9}^{level}$		22.2	54.2	85.1
	${RES}_{10}^{level}$		14.8	22.7	27.8
	${RES}_{10}^{GM}$		164	239	288
	${RES}_{10}^{nGM}$		16.2	25.1	30.8
**Total resistance [**10^8^ *Pa ⋅ s/m*^3^**]**	*RES*	*WG* = 25	74.6	142	209
		*WG* = 30	34.7	65.9	96.9
**Cerebral blood flow [***ml/min*/100 *g***]**	*CBF*	*WG* = 25	16.5	8.64	5.87
		*WG* = 30	19.9	10.5	7.11

**Fig 2:**
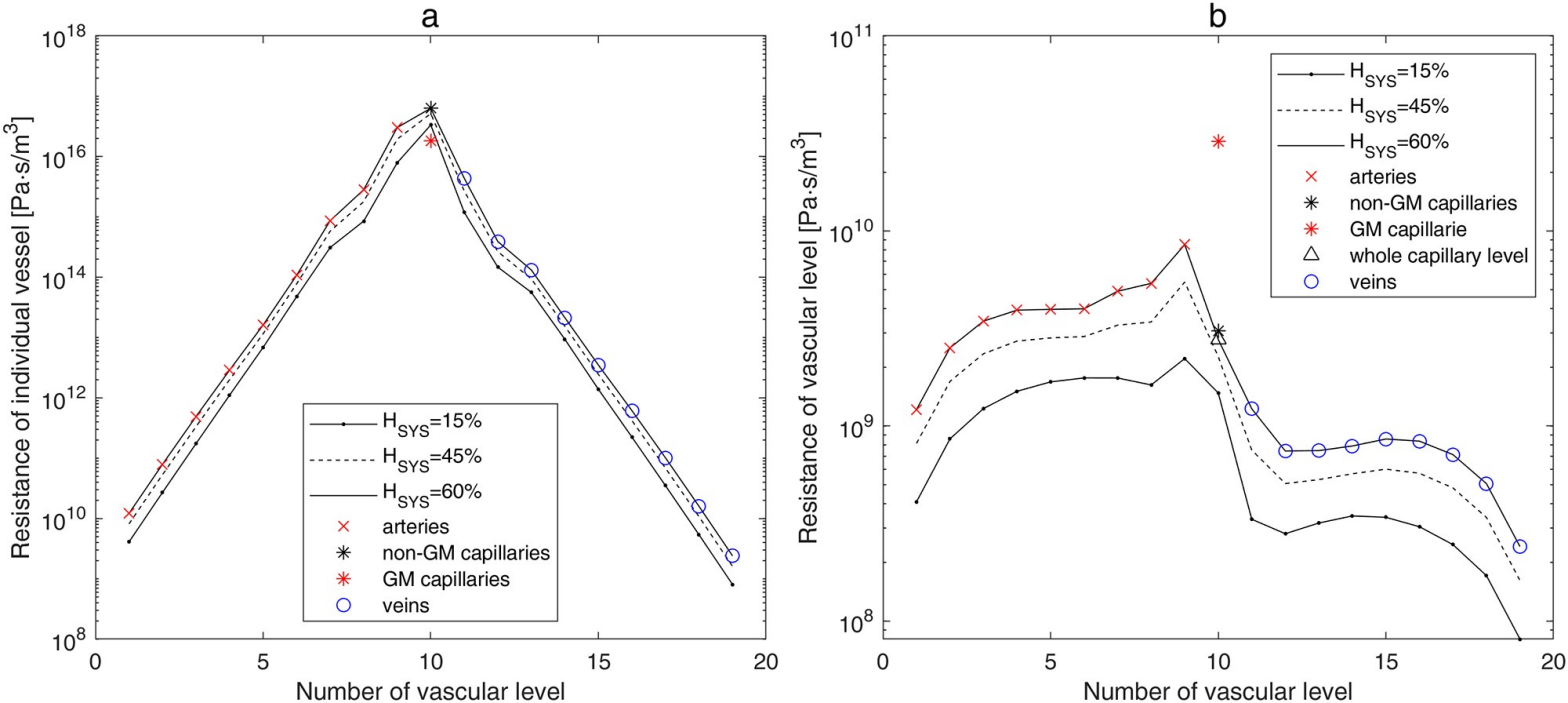
Calculated resistance of the individual vessel (a) and of the whole vascular level (b) for different values of systemic hematocrit.

The values of the apparent viscosity with accounting of *H*_*SYS*_ and individual vessel diameter *d*_*j*_ (Fig 1a) are used for the calculation of the individual vessel resistance *RES*_*j*_ on each level *j* of the hierarchical cerebrovascular model (Fig 2a). The resistance of individual vessels rises with an increase in hematocrit and a decrease in diameter. The resistances of *GM* and non-*GM* capillaries are computed with the two-phase fluid model using tube hematocrit *H*_*T*_. The latter is calculated with accounting for Fåhraeus effect resulting in the reduction of the *H*_*T*_ value with respect to the *H*_*SYS*_ value, which is shown in Fig 1b. The resistance of a single *GM* capillary is lower than that of a non-*GM* capillary (Table 2) because of the difference in diameters (*GM* capillaries have larger diameter than non-*GM* ones).

The total resistance of the whole vascular level ${RES}_{j}^{level}$ in the hierarchical cerebrovascular model depends on the resistance of a single vessel *RES*_*j*_ and the number of the vessels on every level *j*. The resistances ${RES}_{j}^{level}$ calculated for 25 *WG* are shown in Fig 2b. The largest resistance is obtained for the precapillary layer (*j = 9*) with the smallest arterioles. On the capillary level, there is considerable difference in two parallel connected regions (*GM* and non-*GM*) regarding the number of vessels and, consequently, different resistances. Namely, the *GM* has a lower number of vessels and a higher resistance than the rest (non-*GM)* brain region. In our example of a preterm infant with 25 *WG* (Table 2), the increase in hematocrit from *H*_*SYS*_ = 15% (*H*_*T*_ = 10.8%) to *H*_*SYS*_ = 45% (*H*_*T*_ = 36.9%) results in the increase of the ${RES}_{10}^{GM}$ from 163.9 ⋅ 10^8^ to 238.6 ⋅ 10^8^*Pa* ⋅ *s*/*m*^3^ and of the ${RES}_{10}^{nGM}$ from 16.2 ⋅ 10^8^ to 25.1 ⋅ 10^8^*Pa* ⋅ *s*/*m*^3^, which is in good agreement with values presented by [17]. For higher values of *H*_*SYS*_ the total resistance of the vascular levels continues to rise (Fig 2b).

**Fig 3:**
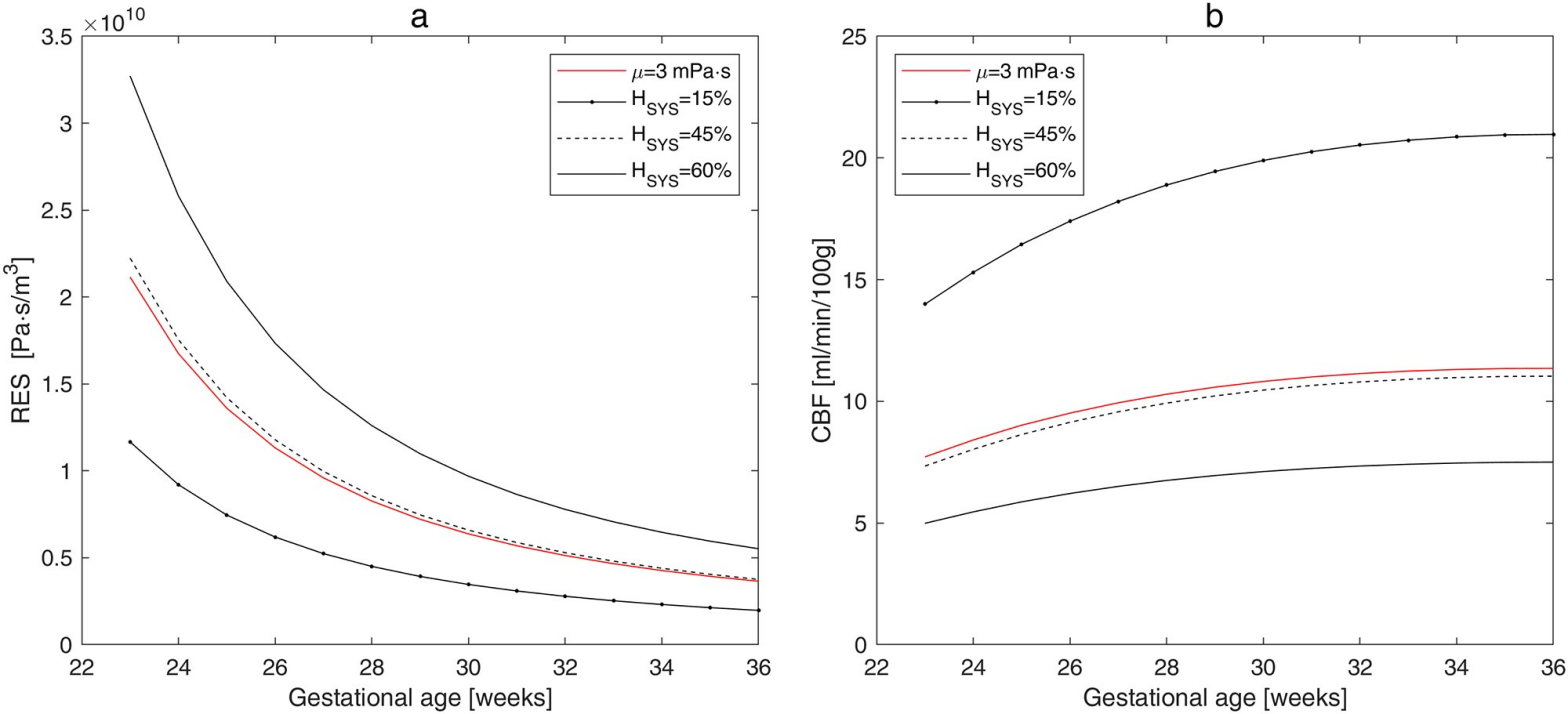
Calculated total cerebral resistance (a) and *CBF* (b) versus gestational age for different values of systemic hematocrit. The red lines show the values calculated using the constant blood viscosity *μ* = 0.003 *Pa* ⋅ *s*.

The effect of hematocrit on the total cerebrovascular resistance *RES* and *CBF* is shown for gestational ages ranging from 23 to 36 *WG* in Fig 3 and for *WG* = 25 and *WG* = 30 in Table 2. Both the reduction of hematocrit and increase of gestational age cause a decrease of the total vascular resistance, resulting in an increase of *CBF*. For all gestational ages, the reduction of *H*_*SYS*_ from 60% to 45% results in 1.5-fold increase of *CBF*; further reduction of *H*_*SYS*_ from 45% to 15% results in 1.9-fold increase of *CBF*.

**Fig 4:**
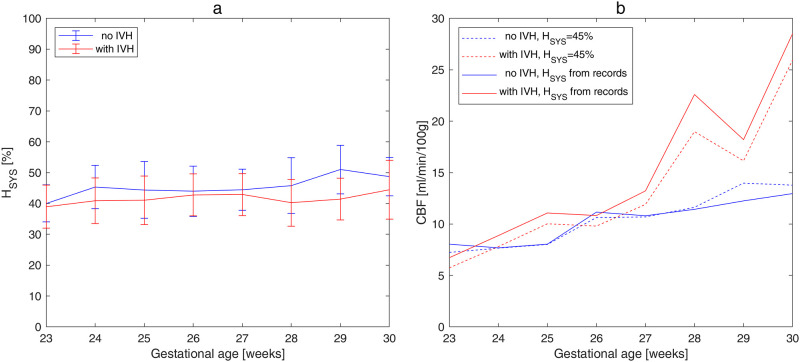
Mean values with standard deviations averaged over gestational age. (a) Measured systemic hematocrit. (b) *CBF* calculated for *H*_*SYS*_ = 45% (dashed lines) and for *H*_*SYS*_ from clinical records (solid lines).

The model developed was applied to clinical records of 254 preterm infants with gestational age ranged from 23 to 30 *WG*. Statistical analysis revealed that the demographic factors did not affect the hematocrit (Table 3). Moreover, the mean hematocrit in the control group (Table 3, no *IVH*) was close to the value of 45% usually taken for numerical calculations (Fig 4a). Therefore, no significant changes (Table 4) in the calculated *CBF* for the control group were obtained due to including measured hematocrit in the mathematical model (Fig 4b). However, the mean value of the hematocrit in the affected group was significantly lower than that in the control group (Table 3). As a result, *CBF* calculated using the measured hematocrit was higher in the affected group (Fig 4b) and this increase was significant for *IVH* grades III+IV (Table 4). This effect was even more noticeable for capillary vessels (Table 5): the increase of *CBF* in individual capillary was statistically significant not only for severe *IVH* (grades III-IV), but already for moderate *IVH* (grad II).

**Table 3:** Dependence of the hematocrit on basic demographic characteristics and *IVH* diagnosis.

Parameter	no	yes	*p-*value
**Male**	42.80 ± 7.49	42.70 ± 8.22	0.72
**Multiple birth**	42.68 ± 7.59	42.93 ± 8.39	0.72
**In Vitro Fertilization**	42.85 ± 7.90	41.78 ± 7.14	0.95
**Natural birth**	42.75 ± 7.98	42.82 ± 6.82	0.65
***IVH***	45.14 ± 7.92	41.41 ± 7.48	0.01

**Table 4:** Global *CBF* [*ml/min*/100 *g*] for different values of *H*_*SYS*._

*IVH*	*H*_*SYS*_ = 45%	*H*_*SYS*_ from records	*p-*value
**No (n = 118)**	9.48 ± 4.57	9.53 ± 4.95	0.79
**All Grades (n = 136)**	11.39 ± 9.24	12.92 ± 11.31	0.004
**Grade I (n = 38)**	12.28 ± 6.28	13.24 ± 7.7	0.53
**Grade II (n = 42)**	10.87 ± 10.98	12.23 ± 12.62	0.09
**Grade III+IV (n = 48+8)**	11.45 ± 8.85	13.22 ± 11.37	0.016

**Table 5:** *CBF* in individual capillary [10^−6^ *ml/min*/100 *g*] for different values of *H*_*SYS*._

*IVH*	*H*_*SYS*_ = 45%	*H*_*SYS*_ from records	*p-*value
**No (n = 118)**	1.48 ± 0.63	1.49 ± 0.69	0.77
**All Grades (n = 136)**	1.49 ± 0.80	1.68 ± 1.00	<0.001
**Grade I (n = 38)**	1.62 ± 0.75	1.78 ± 1.02	0.36
**Grade II (n = 42)**	1.48 ± 0.85	1.64 ± 0.98	0.007
**Grade III+IV (n = 48+8)**	1.46 ± 0.78	1.66 ± 1.01	0.002

## Discussion

Blood hematocrit is an important factor influencing apparent blood viscosity, vessel resistance and blood flow [33, 34]. In the present work, the previously developed hierarchical cerebrovascular model for *CBF* calculation [10, 11] was enhanced by including effects of inhomogeneous hematocrit and vessel diameter on the apparent blood viscosity, vessel resistance and hence *CBF*. The dependence of the apparent viscosity on blood hematocrit and vessel diameter was modeled according to the “*in vivo* viscosity law” derived from experimental data [18]. Calculated values of apparent viscosity were used for the computation of the individual vessel resistance on each level of the hierarchical cerebrovascular mode. Additionally, two-phase fluid model [17] was used for the calculation of the resistance of *GM* and non-*GM* capillaries.

In mathematical modeling performed in the present study, the reduction of hematocrit caused a decrease in the apparent blood viscosity and vascular resistance, resulting in an increase of *CBF*. Our numerical results are in good agreement with values from literature. The apparent blood viscosity calculated for different hematocrit values and vessel diameters were close to the experimental data presented by [12] and the observations by [35] showing twofold increase of the apparent viscosity with an increase of hematocrit from 40% to 60%. In our calculations for vessels with diameter 10 *μm*, a hematocrit increase from 45% to 60% resulted in 1.67-fold increase of the apparent viscosity (see Table 2).

The apparent viscosity of the individual vessel was used for the calculation of total cerebrovascular resistance that depends on the resistance of the individual vessel and number of vessels on each vascular level. In our model, the largest resistance was obtained for the pre-capillary layer with the smallest arterioles, which is consistent with the previous observations [4, 36, 37]. On capillary level, the concentration of *RBC*s near the center of the capillary [13] results in the reduction of hematocrit to a value known as tube hematocrit. We used tube hematocrit values estimated from the systemic hematocrit [32] for the calculation of vascular resistances for two brain regions, namely, the *GM* and the rest, non-*GM*, part of the brain [17]. The *GM* and non-*GM* resistances calculated in our study for 25 *WG* (Table 2) showed the same dependence on hematocrit as in [17].

Mathematical modeling revealed an inverse relationship between *CBF* and hematocrit, which is consistent with experimental studies [37, 38]. A regression analysis based on the xenon-133 measurements of *CBF* in 15 preterm infants with mean gestational age 31 *WG* [38] has demonstrated a significant inverse correlation between *CBF* and hematocrit with the slope -2.3 for the hematocrit variation from 24% to 48%. These results are similar to the 1.9-fold *CBF* increase caused by the hematocrit decrease from 45% to 15% demonstrated for *WG* = 30 in the present study.

The enhanced mathematical model was applied to clinical data collected retrospectively from medical records of 254 preterm infants with gestational age 23–30 weeks. Including the clinically measured hematocrit in the model led to an increase of calculated *CBF* in preterm infants with *IVH* diagnosis. The increase was statistically significant for severe *IVH* (grade III+IV). These results are in agreement with observations that a relatively low hematocrit during the first 24 hours of life correlates with a high incidence of *IVH* [39, 40] and is associated with a prolonged bleeding time [41]. Another important result was the increase of *CBF* in capillary vessels, which was statistically significant for preterms with moderate (grad II) and severe (grad III+IV) *IVH*. Increased *CBF* values in capillary vessels may explain experimental observations that hemorrhage in immature brain often originates from capillary bed or in capillary-vein junction [42]. The exact location of vessel rupture is still under discussion [43] and cannot be determined by the mathematical model used in this study. Thus, the mathematical model developed has demonstrated a correct relationship between decreased hematocrit and enhanced risk of *IVH* in preterm infants.

The following limitations need to be addressed. Whereas some experimental observations demonstrate that *RBCs* aggregation into clusters [35] contribute to the viscosity reduction, these effects were not simulated in the present study. In addition, the influence of the plasma viscosity was not investigated, although some data suggest that plasma viscosity may be more important in the regulation of *CBF* than whole blood viscosity [33]. The effects of *RBCs* aggregation and increased plasma viscosity on *CBF* are considered for further development of the mathematical model for *CBF* calculation.

## Conclusions

The mathematical model for *CBF* calculation has been developed further, by accounting for the effect of inhomogeneous hematocrit on the apparent blood viscosity and vessel resistance. The model is in good agreement with published experimental results. It has been shown that including the clinically measured hematocrit in the mathematical model strongly effects the apparent blood viscosity, vessel resistance and hence *CBF*. Furthermore, in the group of preterm infants with *IVH* diagnosis, the inclusion of measured hematocrit values resulted in a statistically significant increase of calculated *CBF* values compared to calculations with a constant hematocrit value. Thus, accounting for the effect of hematocrit on *CBF* may improve clinical monitoring of preterm infants and prediction of *IVH* development.
